# Crystalloid Solutions in Hospital: A Review of Existing Literature

**DOI:** 10.7759/cureus.39411

**Published:** 2023-05-23

**Authors:** Viraj Panchal, Barath Prashanth Sivasubramanian, Vikramaditya Samala Venkata

**Affiliations:** 1 Medicine, Smt. Nathiba Hargovandas Lakhmichand (NHL) Municipal Medical College, Ahmedabad, IND; 2 Infectious Diseases, University of Texas Health Science Center at San Antonio, San Antonio, USA; 3 Internal Medicine, ESIC Medical College & PGIMSR, Chennai, IND; 4 Department of Internal Medicine, Cheshire Medical Center, Dartmouth-Hitchcock, Keene, USA

**Keywords:** plasma-lyte, ringer lactate, normal saline, sepsis/sirs, pancreatitis, intravenous fluid, crystalloid, critically ill

## Abstract

Intravenous fluids (IVF) like normal saline (NS) and Ringer's lactate (RL) are often crucial in the management of hospitalized patients. Mishandling these fluids can lead to complications in about 20% of patients receiving them. In this review, we present the current evidence through the identification of observational studies and randomized trials that observed the optimal use of IVF. We found that NS may cause hyperchloremic metabolic acidosis in surgical patients, but there is no clear difference in mortality and long-term outcomes between NS and balanced crystalloids. Critically ill patients, particularly those in sepsis, benefit from balanced crystalloids, as high chloride content fluids like NS increase the risk of complications and mortality. In pancreatitis, NS has been shown to increase the risk of ICU admission when compared to RL; however, there is no significant difference in long-term outcomes and mortality between the fluids. RL is preferred for burns due to its isotonicity and lack of protein, preventing edema formation in an already dehydrated state. Plasma-lyte may resolve diabetic ketoacidosis faster, while prolonged NS use can lead to metabolic acidosis, acute kidney injury, and cerebral edema. In conclusion, NS, RL, and plasma-lyte are the most commonly used isotonic IVF in the hospital population. Incorrect choice of fluids in a different clinical scenario can lead to worse outcomes.

## Introduction and background

Water is required for the functioning of every cell in our bodies. Intravenous (IV) fluids such as 0.9% normal saline (NS) or Ringer's lactate (RL) are crucial in the management of critically ill patients. A significant proportion of patients in the hospital require maintenance with intravenous fluids as either a preventative or a corrective measure of their condition [1-3]. It is not reported as much, but mismanagement of fluid therapy can lead to patient harm. As many as one in five patients receiving intravenous fluids and electrolytes suffer complications or morbidity due to their inappropriate administration [4]. While there is an ongoing discussion on the proper use of IV fluids, errors in their use lead to electrolyte emergencies such as hyponatremia and hyperkalemia, both in surgical and medical wards [5,6]. Studies have shown that when IV fluids are used, the provider has little knowledge about their composition or the electrolyte needs of an individual patient [7]. Thus, understanding the literature on managing hospitalized patients using IV fluids is a must, especially when we have unclear evidence for certain clinical situations.

## Review

Types of intravenous fluids

Broadly, intravenous fluids have been classified into three major categories depending on their tonicity, specifically, isotonic, hypotonic, and hypertonic in comparison to water. Isotonic fluids have tonicity that matches the intracellular fluids and are osmotically equal in a range of 240 to 340 mOsm/kg, so there are no significant fluid shifts between intra and cellular space; this allows us to increase the intravascular volume by administration of isotonic fluids. Hypotonic fluids, having less osmotic pressure, force water into the cells from extracellular fluids. Hypertonic fluids, having a higher tonicity than intracellular fluids, draw water out of the cells [8]. Table 1 shows the types of fluid included in each.

**Table 1:** Types of intravenous fluids according to tonicity

Hypotonic	Isotonic	Hypertonic
5% Dextrose	0.9% normal saline	3% Sodium Chloride
0.45% Saline	Ringer Lactate (RL)/Ringers	-
-	Plasma-lyte	-
-	NaHCo3 150 meq in D5% 1000 ml	

Importance of isotonic fluids over others

As isotonic fluids avoid causing significant intracellular fluid shifts and allow us to increase intravascular volume, they are used for a variety of clinical conditions in the hospital. They are used for increasing the intravascular volume in conditions like dehydration, hypotension, sepsis, etc. [9,10]. Since iatrogenic hyponatremia is associated with hypotonic intravenous fluids, isotonic intravenous fluids are favored for a variety of disorders [6]. It may be due to the concentration of sodium that's present in the isotonic fluid that is equivalent to that of plasma, which in turn reduces the possibility of hyponatremia [11]. However, it should be regarded that isotonic fluids may cause hypernatremia and also fluid overload [12].

The two main isotonic fluids used most frequently are normal saline and Ringer's lactate solution. There is a paucity of awareness or evidence about the benefits of specific types of intravenous fluid over others. With this aim, we carried out this review on which isotonic intravenous fluid to choose when managing a patient with a certain condition.

Normal saline

Normal saline is the most commonly used isotonic crystalloid intravenous fluid in the hospital. Normal saline consists of 154 mEq of sodium in each liter bag. It has a tonicity of 308 mOsmol/iter. So while it is used as an isotonic fluid, it is slightly hypertonic compared to plasma (270-300 mOsm/liter).

Normal saline is frequently used in medical treatment and administration, whether it be in hematology or transfusion medicine. As it is isotonic, it is primarily in the interstitial compartment and has the tendency to maintain the expansion of intravascular volume by remaining in the body for a longer period of time [13-15]. However, recently new data shows that it can have adverse effects when compared to a balanced, buffered solution like plasma-lyte or Ringer's lactate [16]. Normal saline has a concentration of 154 mEQ/L of sodium and chloride, with a pH of 5.8 and an osmolarity of 308 mOsm/L [17]. Normal saline in several conditions can cause hyperchloremic, non-anion gap metabolic acidosis, secondarily to an increased amount of chloride which eventually may cause renal vasoconstriction and leads to a decreased glomerular filtration rate (GFR) [18,19]. Many studies have shown that normal saline due to its chloride content has been associated with acute renal failure as well as in-hospital mortality [20,21]. Due to the chloride content, plasma-lyte or Ringer's lactate is preferred over normal saline in critically ill patients suffering from septic shock, acute respiratory failure, gastrointestinal bleeding, acute liver failure, drug toxicity or ingestion, and glucose disorders [2].

Ringer's lactate

While selecting an isotonic fluid agent, due to the association of normal saline with hyperchloremic, metabolic acidosis, there might be a preference towards balanced solutions, which have included acetate, lactate, malate, or even citrate, which helps to metabolize the anions. The most widely used such balanced solution is Ringer's lactate, with a pH limited to a range of 6.5, sodium concentration of 130 mEq/L, and a lower concentration of 109 mEq/L for chloride concentration compared to normal saline, bringing the osmotic concentration to 273 mOsm/L [17]. Thus, one of the drawbacks of normal saline which is of having a higher concentration of chloride is resolved by Ringer's lactate. It has been seen that balanced fluids are associated with a lower reduction in renal blood flow when compared to normal saline, by maintaining the GFR and renal perfusion [22]. Balanced fluids have tonicity similar to plasma making it a more balanced and patient-centered solution theoretically, although its clinical implications are still being explored [23-25]. However, when infused in the body, there has been reported evidence that Ringer's lactate can lead to a decrease in plasma osmolality, thus mimicking a hypotonic solution with a capacity to amplify the water in the brain and increasing the risk of cerebral edema [26]. Hence it is advised to avoid the use of it in patients who are at risk of brain injury or those who are suffering from meningitis, brain tumor, or stroke [18,19,27,28]. Also, Ringer's lactate does not cause a significant increase in lactate concentration when compared to normal saline and hence, those patients with elevated lactate levels can be given Ringer's lactate as an intravenous fluid [29,30].

Plasma-lyte

Plasma-lyte, being from a balanced crystalloid solution, has similar characteristics and benefits to Ringer's lactate over normal saline. Plasma-lyte also has a good buffering capacity to counteract acidosis as it contains a large number of different anions like lactate, gluconate, and acetate which gets converted to bicarbonate, carbon dioxide, and water, eventually addressing the electrolyte deficiency occurring in acidosis [31]. Moreover, plasma-lyte having similar sodium, potassium, and chloride concentration that of plasma and calcium deficit at the same time has an advantage over normal saline for correcting the base deficit in an injured patient after 24-hour intervals [32].

Clinical implications/fluids of choice for resuscitation/clinical scenarios

Role in Surgery

So far existing evidence shows mixed results when assessing the effectiveness of various intravenous fluids in surgical patients. An observational study by Shaw et al. looking at 31,000 patients (30,000 NS and 926 LR) showed statistically significant lower rates of complications (including renal failure requiring dialysis, and electrolyte disturbances) in the group receiving Ringer's lactate compared to normal saline, there was no significant difference in mortality between both the groups [20]. A randomized controlled trial (RCT) of 44 neurosurgical (craniotomy) patients done by Ankita et al. showed increased rates of hyperchloremic metabolic acidosis with normal saline when compared to plasma-lyte which is a balanced crystalloid [33]. British consensus guidelines on intravenous fluids for adult surgical patients recommend against using normal saline from routine use due to the risk of hyperchloremic metabolic acidosis; this recommendation was based on studies that show evidence of acidosis with normal saline without any clear evidence for harm [16,34-39].

Despite the above studies showing the risk of post-operative with normal saline, other studies have not shown a clear advantage for using a particular type of intravenous fluid. In a study done by Bhagat et al; looking at 90 patients undergoing intracranial surgery, normal saline was associated with hyperchloremic metabolic acidosis, but there was no difference between postoperative complications and hospital length of stay between normal saline and Ringer's lactate [40]. The SOLAR trial done by Maheshwari et al., looking at 8616 patients undergoing colorectal and orthopedic surgery (4187 Ringer's lactate, 4429 normal saline), did not report any difference in in-hospital mortality, postoperative renal, respiratory, and infectious complications between both the groups [41]. The study by Emanuel et al. looking at 500 patients during childbirth found an increased risk of acidosis with normal saline but did not find any significant difference in the incidence of 24-hour maternal postoperative morbidity as well as neonatal outcomes between normal saline and Ringer's lactate [42].

Role in Critically Ill Patients (SIRS/SEPSIS and ICU Patients)

Patients who are diagnosed with systemic inflammatory response syndrome (SIRS) have a hypermetabolic stage and a marker deranged electrolyte balance, hence use of proper intravenous fluid and not exaggerating those electrolyte disturbances is important [43]. Normal saline can be used but along comes the hyperchloremic acidosis associated with it, which sensitizes macula densa and decreases the renal blood flow as well as GFR, accelerating acute kidney injury (AKI) [44-46]. A meta-analysis done by Krajewski et al. looking at 6253 patients in the perioperative or intensive care setting found that crystalloids that have a higher chloride content are associated with a higher chance of AKI, metabolic acidosis, increased ventilation time in patients with sepsis compared to those receiving low chloride crystalloid fluid; this study, however, did not find any difference in mortality between both the groups [47]. Retrospective analysis done by Shaw et al. looking at >100,000 patients with SIRS, fluid resuscitation strategy employing lower chloride loads was associated with lower in-house mortality (1.094, 95% CI, 1.062-1.127) [46]. A retrospective study done by Ragunath et al., looking at 53,448 septic patients from 360 USA hospitals, showed decreased rates of in-hospital mortality with the use of balanced solutions like Ringer's lactate compared to normal saline [48].

Studies looking at the intensive care unit (ICU) population as a whole without a specific diagnosis of sepsis show mixed results, while few studies did not find any difference in terms of mortality, acute kidney injury, and rates of renal replacement therapy requirement between normal saline and balanced crystalloid [49,50], a meta-analysis done by Gonzalez et al., looking at 20,684 ICU patients, showed significantly increased rates of mortality with normal saline when compared to balanced crystalloids [51]. It is noted that the use of normal saline or plasma-lyte had no effect on gastrointestinal tolerance, which is defined as having high gastric residual volume (GRV), diarrhea, or even vomiting, in mechanically ventilated patients who were on nasogastric feeds in the ICU [52]. A caveat in using normal saline for diluting drugs in ICU is that it is associated with ICU-acquired hypernatremia [53].

Role in Burns

In this population, the existing literature is unclear regarding the ideal choice of fluid. Despite limited evidence showing that hypertonic solutions, due to their osmolality can lead to decreased total fluid requirement, edema formation, and lower the risk of abdominal compartment syndrome [54-56], hypertonic saline has been shown to lead to an increased risk of hypernatremia, renal failure, and death [57-59]. The British Burns Association recommends Hartmann's solution (HS; a variation of Ringer's lactate) and LR is the most commonly used fluid in the United States and Canada [59,60]. A study by Bedi et al. shows that for maintenance therapy in burns, Ringer's lactate may not be sufficient enough to maintain the electrolyte requirements, and it's low in glucose content. Due to this DNS can be added to Ringer's lactate for maintenance [61]. In burn patients, no difference was seen between two balanced solutions, Ringer's lactate and plasma-lyte [62]. Despite the theoretical benefits of hypertonic saline in burn patients, more studies are needed to evaluate the safety of HS, and patients receiving HS need to be closely monitored for hypernatremia and renal failure.

Role in Trauma

As trauma patients may require large-volume resuscitation, they are susceptible to metabolic acidosis due to poor perfusion and the accumulation of lactate, metabolic acidosis. In trauma patients, normal saline has been shown to be associated with an increased rate of hyperchloremia at 24-hour post injury and abnormal acid-base status compared to balanced crystalloids in trauma patients [32,63,64]. Despite the use of hypertonic saline by some institutions, a study by Bulger et al. found that the use of hypertonic saline compared with normal saline in trauma patients with hypovolemic shock did not result in superior 28-day survival [65].

Role in Pancreatitis

Existing evidence is not definitive regarding the superior choice of IVF in pancreatitis patients. While multiple meta-analyses show a decreased rate of ICU admission, with Ringer's lactate over NS, they did not show a difference in mortality between both groups [66-68]. Similarly double blinded RCT done by Lee et al., showed decreased rates of ICU admissions with LR use, but did not show any difference in rates of recurrent pancreatitis, organ failure, or mortality between both the groups [69] and RCTs done by Wu et al. and Madaria et al. showed decreased rates of inflammation with Ringer's lactate use, as evidenced by decreased rates of SIRS development and decreased levels of inflammatory markers [70,71]. A retrospective study done by Lipinski et al. on 103 patients did not show any difference in length of hospital stay and mortality between NS and LR patients [72].

Role in Special Procedures

A commonly known use of normal saline is to give it with mannitol in patients who are undergoing chemotherapy with cisplatin to prevent damage to the nephrons [73]. But when considering some procedures it becomes important to classify those as which fluid to use. For an irrigating fluid used in bipolar transurethral resection of the prostate (TURP) studies did not find out any superiority for normal saline over Ringer's lactate [74]. While Ringer's lactate is usually a preferred fluid for nasal douching after endoscopic sinus surgery as it is shown to improve postoperative pain and sinonasal symptoms, which was shown in a study comparing it with normal saline [75]. Compared to isotonic and hypertonic saline, Ringer's lactate solution may produce less noxious stimuli within the nasal mucosa and improve mucociliary function, resulting in improved outcomes [75]. The amount of fluid used also does have an important role. One study did not find a difference in the routine use of high- or low-volume use of plasma-lyte to prevent hypotension and other complications in adults who are undergoing elective colonoscopy [76].

When considering certain procedures, warm normal saline irrigation used in ureteral endoscopic surgeries often results in better surgical outcomes like having a lower ureteral spasm rate with a greater relaxation of the ureteral muscle. It also showed lower chances of having various complications, like impaction of the ureteroscope, dislodgement, or retropulsion of ureteral stone, compared to cold normal saline irrigation [43]. To reduce postoperative pain, the installation of normal saline intraperitoneally following laparoscopic cholecystectomy is a good choice for reducing postoperative pain when compared to the administration of analgesics after six hours and after 12 hours following the procedure [77]. Also, postoperative outcomes in terms of wound infection and length of hospital stay are better when normal saline is used to irrigate subcutaneous tissue under pressure for appendicitis wound closure as compared to the standard protocol [78].

It was also observed that the so-called placebo effect for giving an intra-articular normal saline injection did show a meaningful response clinically in patient-reported outcomes till months with knee osteoarthritis when compared to hyaluronic acid in the treatment group [79]. Similarly in myofascial pain syndrome, injection of normal saline at trigger points was preferred over the conventional drug mixture (lidocaine 1% 10 mL + triamcinolone acetonide 40 mg/mL), which was found useful not just because of its financial advantage but also had a more acceptable side effect profile compared to conventional therapy [80]. Also, local injection of normal saline has demonstrated more improvement clinically in terms of pain and also functional outcomes in those who are suffering from lateral epicondylitis when compared to placebo [81]. In patients in whom allergic rhinitis is suspected, nasopharyngeal irrigation with normal saline has been shown to better relieve symptoms compared to the conventional nasal corticosteroid application [82]. Likewise, when compared to vaginal misoprostol, infiltration of normal saline has been found to be a good alternative at the time of surgery which again is associated with lesser complications and a better efficacy with taking a shorter time to dilate the cervix [83]. When spontaneous respiration failed to occur in patients with tonsillar herniation even after external ventricular drainage or when the supratentorial lesion was removed, at that time clinical usefulness of normal saline injection via lumbar puncture helped regain spontaneous breathing and full recovery in 24.4% of the participants. The overall effectiveness of this procedure was found to be 60% [84]. However, not all procedures can replace the conventional treatment, because local anesthetic with adrenaline is always preferred for septoplasty over normal saline [85].

The foremost thing while placing a chest tube is to seal the track properly to prevent the development of pneumothorax after inserting a chest tube for any procedure, especially taking lung biopsy either via percutaneous transthoracic route or CT-guided, and it was seen that sealing the track with normal saline is a straightforward way to reduce the incidence of its associated complications [86,87].

Considering the use of normal saline, one also needs to know that it could be used to measure intra-abdominal pressure, especially in critically ill patients where it is measured intravesically. Normal saline is associated with accurate results [88]. Furthermore, injecting 5 ml of normal saline just before administering hyperbaric bupivacaine is an effective way to prevent postural puncture headaches in patients with cesarean section [89]. Using a normal saline wash to remove all the local anesthetic from a nerve block especially to prevent diaphragmatic paralysis did not show a decrease in the level of analgesia. But the use of normal saline showed a significant reduction in full hemidiaphragmatic paralysis, as most of the progression of the drug was halted which resulted in only partial paralysis [90]. 

Role in Diabetic Ketoacidosis

Diabetes can cause a potentially fatal metabolic and homeostatic disorder called diabetic ketoacidosis (DKA). Currently, management for any patient with DKA is to recover the fluid that has been lost, correct the hyperglycemia with insulin, and correct the associated electrolyte losses and any imbalance [91]. The aim of the fluid replacement is to expand the intravascular volume which is primarily obtained with isotonic fluid. There are different factors that may affect the choice of fluid which are the hydration status of the patient, electrolyte levels, and urine output. Normal saline has been used most often as a fluid of choice in DKA patients [92]. However, with recent studies indicating the risk of increased chloride levels, acute kidney injury, and metabolic acidosis with the use of normal saline, there is a concern for similar results affecting DKA patients [2,18,19,93]. Our review showed mixed results with no definitive results as noted below.

A study by Oliver et al. [94] compared plasma-lyte and normal saline and did not find any significant difference between the two fluids in the resolution of DKA, however, they showed that when a large amount of normal saline is administered, the serum chloride level increases. Chua et al. [95] found a significant resolution of DKA when the patients were given plasma-lyte compared to normal saline. They showed that the chloride levels were not markedly elevated in those who received plasma-lyte, and emphasized the factor that hyperchloremia may also be associated with the type of fluid which is given for the resuscitation. Similar results were found in different studies also [96,97]. A randomized double-blinded controlled trial by Van Zyl et al. [98] found that pH normalizes faster if Ringer's lactate is used as a primary resuscitation fluid instead of normal saline, however, the results were not significant and they did not find any difference in the rate of resolution of DKA. When considering the resolution of DKA, one tries to look at the evidence of the disappearance of ketoacid anions, which is reflected by the serum anion gap becoming less than 12 mEq/L. The anion gap takes into consideration of beta-hydroxybutyrate and also provides reasonable evidence of changes that occur during resuscitation. Thus normalization of the anion gap helps to know that ketoacid anions have disappeared from the serum. When volume expansion occurs through intravenous resuscitation, loss of ketoacid anions occurs through renal excretion and hyperchloremic acidosis soon resolves as the kidney is able to excrete ammonium chloride ions and at the same time regenerate bicarbonate ions [90-101]. There are two different mechanisms by which Ringer's lactate is cleared. In normal individuals, lactate undergoes gluconeogenesis and results in elevated levels of blood glucose which gets corrected with insulin, however, this does not occur in DKA patients [102]. Secondly, lactate is oxidized to carbon dioxide and water. Hydrogen ions are consumed in both gluconeogenesis and water, which helps in lowering acidosis in DKA patients [103]. This might explain the faster normalization of pH with Ringer's lactate compared to normal saline in the study done by Van Zyl et al. [98] however normalization pH does not imply that ketoacid anions have been renally excreted.

Even though studies indicate a possible risk of worsening metabolic acidosis, and increased chloride levels with normal saline, current evidence is not definitive that the use of balanced crystalloid solutions like LR or plasma-lyte will lead to improvement in rates resolution of DKA and mortality. More prospective studies are needed to determine the ideal choice of crystalloid fluid in DKA patients.

Role in Kidney Transplant

In patients with acute or chronic end-stage kidney disease, a transplant should be considered over dialysis because it improves the quality of life. Crystalloids are routinely used during kidney transplantation to maintain optimal intravascular volume, which is essential for graft perfusion and function [104-107]. During transplantation and after establishing vascularity in the transplanted kidney, it is important to maintain appropriate electrolyte levels and acid-base balance. Therefore, the choice of fluid becomes important in preventing electrolyte abnormalities and acid-base disorders [108]. Plasma-lyte or normal saline, either of each can be used in patients with uncomplicated living donor kidney transplantation, but plasma-lyte does show better acid-base and electrolyte balance compared to normal saline, most importantly in the post-reperfusion period also [108]. Patients receiving normal saline were having higher chances of hyperkalemia and hyperchloraemia, and were more prone to become acidotic compared to those receiving plasma-lyte postoperatively [107]. Some also showed delayed graft functioning in the recipients receiving normal saline perioperatively [109]. However, the role of hyperchloremic acidosis was not associated with delayed graft function, suggesting an alternative chloride-independent mechanism [110]. A prospective study done by Saini et al. [111] compared the use of normal saline, Ringer's lactate, and plasma-lyte in 60 renal transplant patients in each group and also found that balanced crystalloid fluid was better than normal saline. Another study that compared all three fluids with each other done by Hadimioglu et al. [112] also found that normal saline was associated with a significantly increased amount of chloride and caused a decrease in pH. Among balanced solutions, Ringer's lactate was found to be significant in increasing blood lactate levels. However, no significant change in pH or lactate levels were seen in those patients who received plasma-lyte during kidney transplant [112]. Similar results were shown by Kim et al. [108], Modi et al. [113], and O'Malley et al. [34]. Therefore, current evidence suggests plasma-lyte might be superior to normal saline for kidney transplantation, but more large randomized controlled studies are needed to definitively assess the differences. 

There was a statistically significant decrease in pH (7.44 +/- 0.50 vs 7.36 +/- 0.05), base excess (0.4 +/- 3.1 vs -4.3 +/- 2.1), and a significant increase in serum chloride (104 +/- 2 vs 125 +/- 3 mM/L) in patients receiving saline during surgery. Lactate levels increased significantly in patients who received Ringer's lactate (0.48 +/- 0.29 vs 1.95 +/- 0.48). No significant changes in acid-base measures or lactate levels occurred in patients who received plasma-lyte. Potassium levels were not significantly changed in any group.

## Conclusions

Ringer's lactate, normal saline, and plasma-lyte are the most commonly used isotonic intravenous fluids in the hospital population. Incorrect choice of fluids in a different clinical scenario can lead to worse outcomes. There is a paucity of knowledge among providers about the various advantages and disadvantages of various fluid choices.

In surgical patients, normal saline was associated with the development of hyperchloremic metabolic acidosis but current evidence does not show any difference in mortality and long-term outcomes between normal saline and balanced crystalloid solutions. Metabolic acidosis needs to be taken into consideration before selecting intravenous fluids in perioperative patients.

In sepsis/sirs patients, existing evidence strongly shows an increased risk of complications, including increased risk of mortality with high chloride content intravenous solutions (normal saline), when compared to balanced crystalloids. When studies look at the ICU population as a whole, without a specific diagnosis of sepsis, studies do not show a difference in mortality and acute complications between normal saline and balanced crystalloid solutions. This reiterates the importance of favoring balanced crystalloid solutions for sepsis patients.

With pancreatitis, while multiple studies and large meta-analysis suggests an increased risk of SIRS development and ICU admission with normal saline when compared to Ringer's lactate, current evidence does not show a difference in mortality and long-term outcomes between normal saline and balanced crystalloids. More studies are needed to assess if a particular fluid choice is superior to other types of fluids.

Ringer's lactate solution is the preferred fluid for burns and is widely used in the USA and Canada. Hypertonic sodium lactate solution has been found to have adverse effects and it did not decrease total fluid requirements.

Normal saline is commonly used in DKA patients but has associated risks such as metabolic acidosis, acute kidney injury, and cerebral edema. Studies have shown that plasma-lyte may have advantages such as a lower risk of metabolic acidosis and faster resolution of DKA.

Limited evidence suggests that the use of balanced crystalloid solutions in renal transplant patients might lead to lower rates of electrolyte and acid base imbalances, but more studies are needed to assess which fluid choice is superior.
